# A Novel Porcine Model of Ischemia-Reperfusion Injury After Cross-Clamping the Thoracic Aorta Revealed Substantial Cardiopulmonary, Thromboinflammatory and Biochemical Changes Without Effect of C1-Inhibitor Treatment

**DOI:** 10.3389/fimmu.2022.852119

**Published:** 2022-04-01

**Authors:** Erik Waage Nielsen, Yoav Miller, Ole-Lars Brekke, Joost Grond, Anh Hoang Duong, Hilde Fure, Judith Krey Ludviksen, Kristin Pettersen, Leon Reubsaet, Rigmor Solberg, Harald Thidemann Johansen, Tom Eirik Mollnes

**Affiliations:** ^1^ Department of Anesthesia and Intensive Care Medicine, Nordland Hospital, Bodø, Norway; ^2^ Institute of Clinical Medicine, UiT The Arctic University of Norway, Tromsø, Norway; ^3^ Faculty of Nursing and Health Sciences, Nord University, Bodø, Norway; ^4^ Department of Immunology, Faculty of Medicine, University of Oslo, Oslo, Norway; ^5^ Research Laboratory, Nordland Hospital Trust, Bodø, Norway; ^6^ Faculty of Health Sciences, Kristian Gerhard (K.G.) Jebsen Thrombosis Research Center (TREC), UiT The Arctic University of Norway, Tromsø, Norway; ^7^ Department of Pharmacy, University of Oslo, Oslo, Norway; ^8^ Department of Immunology, University of Oslo and Oslo University Hospital, Oslo, Norway; ^9^ Centre of Molecular Inflammation Research, Norwegian University of Science and Technology, Trondheim, Norway

**Keywords:** complement - immunological terms, Kallikrein - kinin system, porcine (pig) model, C1-INH (C1 inhibitor), C1-INH concentrate, ischemi/reperfusion injury, ANOVA & factor analysis

## Abstract

Ischemic injury worsens upon return of blood and innate immunity including the complement system play a central role in ischemia-reperfusion injury (IRI) as in thoracic aortic surgery. Complement component1 inhibitor (C1-INH) has been shown to reduce IRI and is a broad-acting plasma cascade inhibitor. We established a new porcine model of IRI by cross-clamping the thoracic aorta and evaluated the global changes occurring in organ function, systemic inflammatory response and organ damage with or without treatment with C1-INH-concentrate. Twenty-four piglets (8.8-11.1 kg) underwent 45 minutes clamping of the thoracic aorta at the Th8 level. Upfront 12 piglets received human saline and 12 received C1-INH (250 IU/kg) intravenously. Three sham animals received thoracic opening without clamping. Reperfusion lasted 5 hours. We studied ten cardiorespiratory markers, three hematologic markers, eleven inflammatory markers, and twelve organ damage markers over the whole experimental period. Postmortem tissue homogenates from seven organs were examined for inflammatory markers and analysed by two-way repeated-measures ANOVA, area under the curve or unpaired t-tests. By excluding sham and combining treated and untreated animals, the markers reflected a uniform, broad and severe organ dysfunction. The mean and range fold change from before cross-clamp onset to maximum change for the different groups of markers were: cardiorespiratory 1.4 (0.2-3.7), hematologic 1.9 (1.2-2.7), plasma inflammatory 19.5 (1.4-176) and plasma organ damage 2.9 (1.1-8.6). Treatment with C1-INH had only a marginal effect on the IRI-induced changes, reaching statistical significance only for the plasma complement activation product TCC (p=0.0083) and IL-4 (p=0.022) and INF-α (p=0.016) in the colon tissue. In conclusion, the present novel model of porcine global IRI is forceful with regards to central markers and could generally be applicable for pathophysiological studies. C1-INH treatment had no significant effect, but the model allows for future testing of other drugs attenuating IRI globally.

## Introduction

Ischemia-reperfusion injury (IRI) is tissue damage caused by returning blood to organs temporarily deprived of circulation. IRI arises in coronary and cerebral infarctions, trauma, surgery and transplantation. Global IRI have been studied by clamping the aorta in various rat, rabbit and dog models. The translational value to humans are limited from small animal models and could advantageously be studied in large animals, where pig is a highly relevant animal model.

Several porcine models of thoracic aortic clamping have been described. However, these models have focused on the effect of single organs and not taken into account the global IRI from all organs affected by the clamping. Studies are described for the effects on cardiac (1, 2), renal (3, 4), hepatic (5), limb (6), spinal cord (7), and pulmonary IRI (8).

We aimed to establish a new porcine model for thoracic aortic cross-clamping using light weight piglets to allow for a minimal use of costly experimental therapeutics, while preserving translational value of the model. Such a model is highly relevant since thoracic aortic aneurysm surgery often require clamping of aorta. We have shown that the inflammatory reaction in such patients is largely mediated through the lectin complement pathway (9).

In IRI, reactive oxygen species and innate inflammation exacerbate ischemic injury through e.g. increased complement activation (10). Complement 1 inhibitor (C1-INH) controls the physiological activity of both the classical and lectin pathway of complement, and several studies show that C1-INH reduces IRI in animal models and in patients (10). In addition, C1-INH is by far the most important suppressor of bradykinin production from the kallikrein-kinin system, and would inhibit bradykinin-mediated inflammation in pigs (11), capillary leakage and pain (12).

The aim of the present study was to establish a novel and clinically relevant IRI pig model of aortic clamping. Secondly, to investigate the effect of IRI on cardiovascular, hematologic, inflammatory and organ damage markers during ischemia and for a five hour observation period, and thirdly, how these effects were modified by supraphysiological amounts of human C1-INH administered before clamping.

## Materials and Methods

### Animal Handling

This study was approved by the National Animal Research Authority (FOTS-ID-8197). We performed the experiments under the Norwegian Laboratory Animal Regulations and the EU directive 2010/63/EU. Animals were healthy Specific Pathogen Free Norwegian landrace pigs from one farm close to the University and the operation theatre. The study was led by well-trained staff with over 10 years of experience in porcine anaesthesia. In total, 27 Norwegian Landrace pigs (mean weight: 9.9 ± 0.6 kg), 2 females (one in each treatment group) and 25 males were included in the study. The pigs received intramuscular anaesthesia consisting of 500 mg ketamine, 40 mg azaperone and 0.5 mg atropine blended in a 20 ml injection set at the farm maintaining spontaneous respiration. They were weighed and received one intravenous line in each ear. Anaesthesia was deepened with intravenous ketamine 50-100 mg and continuous infusion of 1 mg/kg/h midazolam and 1.1 µg/kg/h remifentanil. Prior to 45 minutes of aortic clamping, 12 animals received a bolus of saline solution and 12 animals received human C1-INH (250 U/kg). The dose was based on our previous study on the amount C1-INH needed to inhibit the complement activation (13), and other *in vivo* studies (14). Three sham animals received thoracic opening without aortic clamping or infusion. Intravenous boluses of 2-100 ml nitroprusside (200 µg/ml) and 3-5 ml propofol (10 mg/ml) were administered to adjust mean arterial pressure (MAP). After release of the aortic cross-clamp remifentanil was switched to fentanyl 66 µg/kg/h.

### Monitoring

In short, animals were endotracheally intubated and received an arterial line in the left carotid artery, a central venous catheter in the left external jugular vein, a pulmonary catheter in the right external jugular vein and a suprapubic catheter for monitoring and sample collection purposes.

### Aortic Clamping

A mini thoracotomy at the level of Th8 was performed, ribs opened with a Finochietto Retractor and the descending thoracic aorta was exposed. After manual palpation, aorta was occluded with a medium sized Satinsky vascular clamp. Surgery was followed by a stabilization period of 30 minutes. Prior to aortic clamping the MAP was reduced to 30-40 mmHg with help of sodium nitroprusside and propofol bolus doses. In order to complete this severe period of ischemia, it was necessary to use potent vasopressors and vasodilators with quick and large adjustments. After initiation of clamping, total ischemia of the lower extremities was confirmed with an ultrasound Doppler of the femoral artery. The immediate increase in MAP after initiation of clamping was stabilized at 80-100 mmHg during the 45-minute ischemic period using in average 80.8 mg propofol and 13.3 mg nitroprusside. Noradrenaline was initiated after 35 minutes of ischemia to elevate blood pressure before release of the aorta clamp. Tribonate^®^ 30 ml was administered at the 45-minute ischemia mark in anticipation of a fall in pH. The cross-clamp was removed and replaced by digital pressure and both the surgeon and anaesthetist carefully monitored end-tidal CO_2_-values, heart rate and blood pressure. The anaesthetist used noradrenaline bolus doses to control blood pressure at this crucial time. It normally took around 10 minutes before digital pressure could be fully released and vital signs stabilized. The aim at this point was to keep MAP above 50 mmHg. If the dose of noradrenaline infusion reached 3.2 µg/kg/min a continuous infusion of vasopressin was added at a concentration of 1 U/ml with a start rate of 0.5 ml/h. In average 13.35 mg noradrenaline and 7.83 U vasopressin were used. If pH-values dropped below 7.2 boluses of 10 ml Tribonate^®^ were administered, in average 44.6 ml. The average amount of infused Ringer Acetate was 694 ml. Neither of the above mentioned regimens were statistically different between the two groups. Overall, the hemodynamic management was performed in accordance with clinical practice, although experimental treatment of pigs cannot fully mimic the clinical situation.

### Hemodynamic and Respiratory Parameters

Heart rate (HR) and mean arterial pressure (MAP) were measured using an arterial catheter (Millar Instruments, Houston TX). Mean pulmonary artery pressure (MPAP) was measured using a pulmonary catheter (Edwards Lifesciences, Irvine, CA). Animals were ventilated using the Pulmonetic System^®^ LTV 1000 ventilator (Pulmonetic Systems, Minneapolis, MN). Fraction of inspired oxygen (FiO_2_), respiratory minute volume (MV) and peak respiratory pressure were adjusted to normal saturation, pH and end-tidal CO_2_ (ETCO_2_) values when possible. Registration of hemodynamic and respiratory values, as well as blood sampling were done at the following time-points relative to start of reperfusion: -75 minutes (baseline), -50 minutes (before cross-clamp onset), -5 minutes (gradual release of clamp), 0 minutes (start reperfusion), and 60, 120, 180, 240 and 300 minutes after start of reperfusion.

### Blood Analyses

Arterial blood gases and lactate were analysed directly on site using an epoc^®^ Blood Analysis System (Epocal, Ottowa, ON). Haematological parameters were analysed in EDTA-blood on the ADVIA^®^ 2120i System (Siemens healthineers, Eschborn, Germany). Biochemical parameters were analysed in heparinized plasma on the ADVIA^®^ 1800 Chemistry System (Siemens healthineers, Tarrytown, NY). For the analyses of inflammatory and coagulation parameters EDTA and citrated blood samples were cooled and centrifuged at 1500 x g for 15 minutes immediately after collection and the obtained plasma was frozen at -80°C. Cytokines were analysed in EDTA-plasma using a 9-plex assay (eBioscience, San Diego, CA). C3a was measured in EDTA-plasma as previously described in detail (15). Soluble Terminal Complement Complex (TCC) levels were measured in EDTA-plasma using an in-house ELISA (16). Kallikrein activity was measured as measured by cleavage of the peptide substrate S-2302 (Pro-Phe-Arg-pNA) and calculating the increase in O.D. at 405 nm in citrate plasma as previously described (17). PAI-1 was measured in citrate plasma by ELISA (Molecular Innovations, Novi, MI). TAT was measured in citrate plasma (Siemens Healthineers, Newark, DE).

### Cytokines in Tissue Homogenates

After 300 minutes of reperfusion, animals were euthanized by intravenous injection of potassium chloride. Biopsies were harvested from the left ventricle, left lung, liver, left kidney, spleen, small intestine and colon. Biopsies were rinsed in ice-cold saline and snap-frozen on dry-ice prior to storage at -80°C. Biopsies were homogenized and prepared for analysis as described previously (18). Tumour necrosis factor (TNF), interleukin (IL)-1-β, IL-4, IL-6, IL-8, IL-10, IL-12p40, interferon (IFN)-α and IFN-γ-levels were analysed using 9-plex (eBioscience, San Diego, CA).

### Statistical Analyses

GraphPad Prism 8.4.3 (San Diego, CA, USA) was used for result handling and statistical analyses. Longitudinal time data in graphs were expressed as median and interquartile range. Markers of physiological organ function, systemic inflammatory response and organ damage were analysed using two-way repeated measures ANOVA with Geisser-Greenhouse correction and mixed effects analysis in case of missing values, as well as with t-test of the area under the curve calculated in Excel. Cytokine values from tissue homogenates were log-transformed and analysed using independent t-tests. P-values < 0.05 were considered significant.

## Results

A broad panel of markers changed considerably from before cross-clamp onset and showed a uniform pattern in both C1-INH-treated and untreated animals. The variation in time course of each marker is shown. The only marker that was significantly reduced by C1-INH treatment was TCC. To show the magnitude of the total response, we present the fold change (FC) of the markers in treated and untreated animals combined, excluding sham animals. The change is from 50 minutes before the start of IRI (approximately 5 minutes before cross-clamp onset) until maximal change of marker value during the following observation period. FC for each marker is mean of maximal values after clamping in all animals divided by mean value before clamping. Each marker´s name in the Figure panels is succeeded by the FC in parenthesis when meaningful.

### Cardiorespiratory Markers

**Figure 1:**
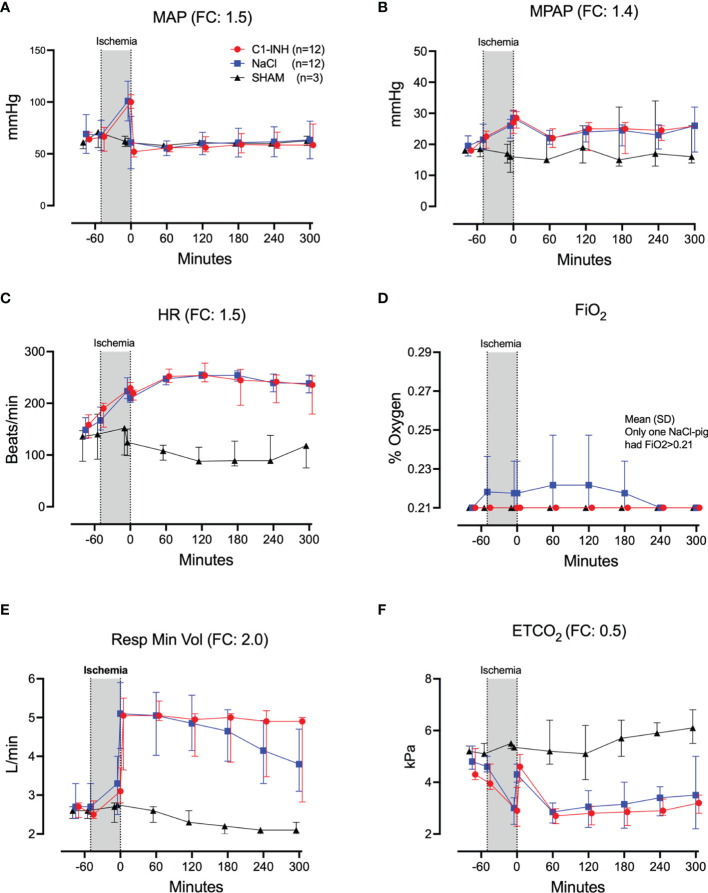
Cardiorespiratory response I. **(A)** Mean arterial pressure (MAP), **(B)** mean pulmonary arterial pressure (MPAP), **(C)** heart rate (HR), **(D)** fraction of inspired oxygen (FiO_2_), **(E)** respiratory minute volume (Resp Min Vol), and **(F)** end-tidal CO_2_ (ETCO_2_) were measured in 24 piglets undergoing 45 minutes of clamping of the thoracic aorta at the Th8 level. Upfront 12 piglets received human saline and 12 received C1-INH (250 IU/kg) intravenously, respectively. Three sham animals received thoracic opening without clamping. Reperfusion started at 0 minutes and lasted 5 hours. Longitudinal time data are expressed as median and interquartile range. “Ischemia” indicates the 45 minutes period of clamping. NaCl and C1-INH-groups were combined when there were no differences in the fold change (FC) calculation. FC is the change from before cross-clamp onset to maximum change.

**Figure 2:**
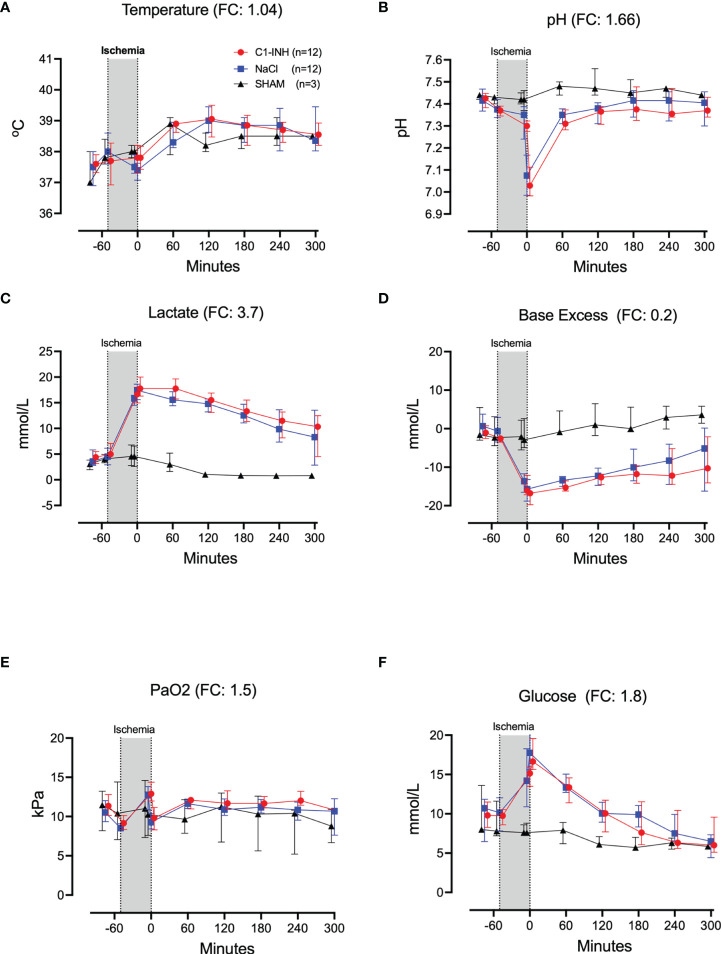
Cardiorespiratory response II. **(A)** Temperature, **(B)** pH, **(C)** lactate, **(D)** Base Excess, **(E)** PaO2, and **(F)** Glucose were measured in 24 piglets undergoing 45 minutes of clamping of the thoracic aorta at the Th8 level. FC for Ph was calculated with absolute numbers of hydrogen ions. Upfront 12 piglets received human saline intravenously and 12 received C1-INH (250 IU/kg), respectively. Three sham animals received thoracic opening without clamping. Reperfusion started at 0 minutes and lasted 5 hours. Longitudinal time data are expressed as median and interquartile range. “Ischemia” indicates the 45 minutes period of clamping. NaCl and C1-INH-groups were combined when there were no differences in the fold change (FC) calculation. FC is the change from before cross-clamp onset to maximum change.

Twelve cardiorespiratory markers were investigated. The absolute values and time course of MAP, MPAP, HR, FiO_2_, Resp Min Vol, and ETCO_2_ are presented in **Figures 1A–F**. In addition, the fold change is presented as text in parenthesis after each marker´s name. Taken together, the mean with range fold change was 1.4(1–2). The temperature, pH, lactate, Base Excess, PaO2 and glucose are presented in **Figures 2A–F**. The fold change is in parenthesis. Taken together the mean with range fold change of these markers was 1.65 (0.2-3.7).

### Hematologic Markers

**Figure 3:**
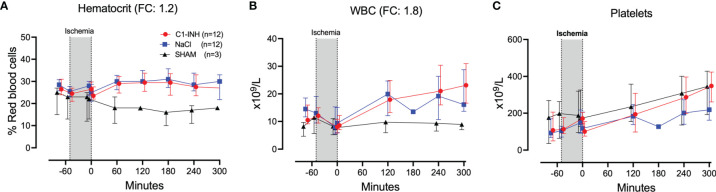
Hematology. **(A)** Hematocrit, **(B)** white blood cells (WBC), and **(C)** platelets were measured in 24 piglets undergoing clamping of the thoracic aorta at the Th8 level. Upfront 12 piglets received human saline intravenously and 12 received C1-INH (250 IU/kg), respectively. Three sham animals received thoracic opening without clamping. Reperfusion started at 0 minutes and lasted 5 hours. Longitudinal time data are expressed as median and interquartile range. “Ischemia” indicates the 45 minutes period of clamping. NaCl and C1-INH-groups were combined when there were no differences in the fold change (FC) calculation. FC is the change from before cross-clamp onset to maximum change.

The absolute values and time course of three hematologic markers are presented in **Figures 3A–C**. The fold change is in parenthesis. Taken together, hematocrit, white blood cells, and platelets had a mean and range fold change of 1.9 (1.2-2.7).

### Inflammatory Markers

**Figure 4:**
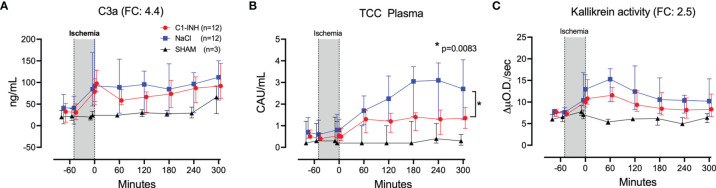
Complement and kallikrein activation. **(A)** C3a, **(B)** terminal C5b-9 complement complex (TCC), and **(C)** kallikrein activity were measured in plasma of 24 piglets undergoing clamping of the thoracic aorta at the Th8 level. Upfront 12 piglets received human saline intravenously and 12 received C1-INH (250 IU/kg), respectively. Three sham animals received thoracic opening without clamping. Reperfusion started at 0 minutes and lasted 5 hours. Longitudinal time data are expressed as median and interquartile range. “Ischemia” indicates the 45 minutes period of clamping. NaCl and C1-INH-groups were combined when there were no differences in the fold change (FC) calculation. FC is the change from before cross-clamp onset to maximum change. * meaning: Probability of randomly seeing such a difference after C1-inhibitor treatment.

**Figure 5:**
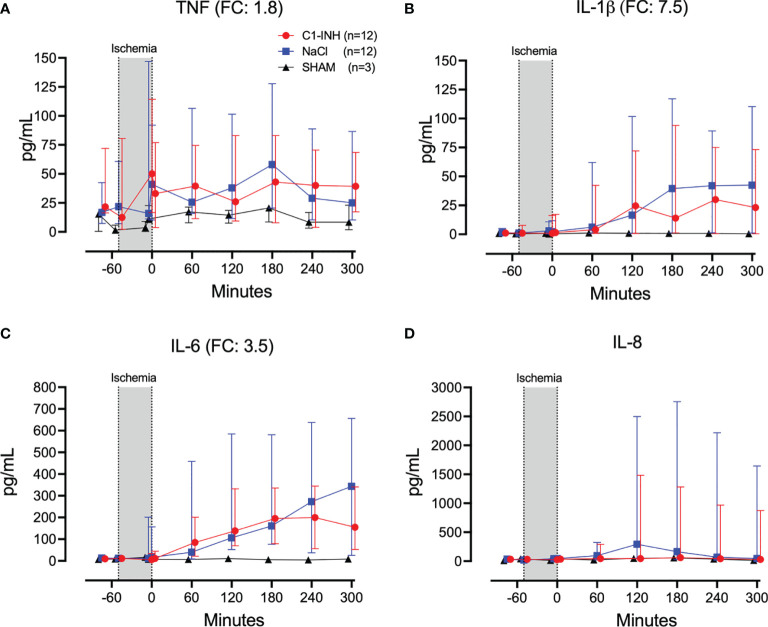
Proinflammatory cytokines. **(A)** Tumour necrosis factor (TNF), **(B)** interleukin (IL)-1β, **(C)** IL-6, and **(D)** IL-8 were measured in plasma of 24 piglets undergoing clamping of the thoracic aorta at the Th8 level. Upfront 12 piglets received human saline intravenously and 12 received C1-INH (250 IU/kg), respectively. Three sham animals received thoracic opening without clamping. Reperfusion started at 0 minutes and lasted 5 hours. Longitudinal time data are expressed as median and interquartile range. “Ischemia” indicates the 45 minutes period of clamping. NaCl and C1-INH-groups were combined when there were no differences in the fold change (FC) calculation. FC is the change from before cross-clamp onset to maximum change.

**Figure 6:**
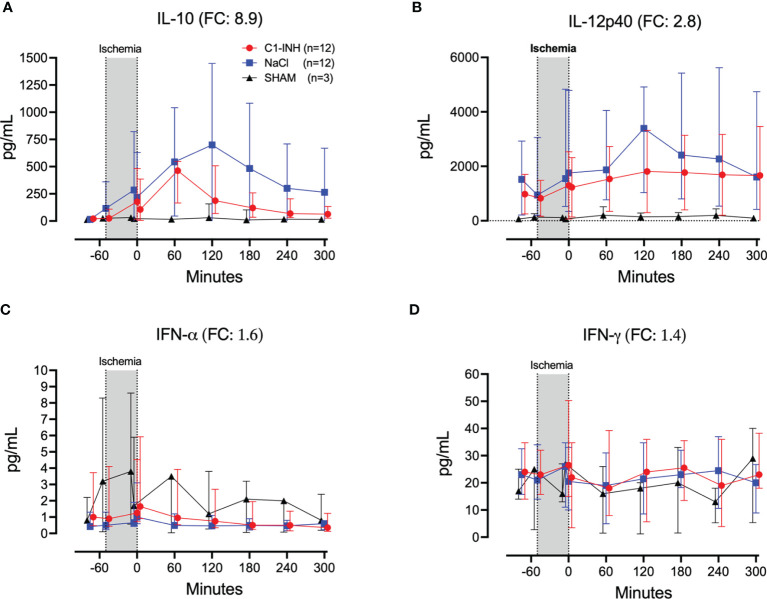
Regulatory cytokines and interferons. **(A)** Interleukin (IL)-10, **(B)** IL-12p40, **(C)** interferon (IFN)-α, and **(D)** IFN-γ were measured in plasma of 24 piglets undergoing clamping of the thoracic aorta at the Th8 level. Upfront 12 piglets received human saline intravenously and 12 received C1-INH (250 IU/kg), respectively. Three sham animals received thoracic opening without clamping. Reperfusion started at 0 minutes and lasted 5 hours. Longitudinal time data are expressed as median and interquartile range. “Ischemia” indicates the 45 minutes period of clamping. NaCl and C1-INH-groups were combined when there were no differences in the fold change (FC) calculation. FC is the change from before cross-clamp onset to maximum change.

Eleven inflammatory markers were investigated. The absolute values and time course of complement activation measured by C3a, TCC and kallikrein activity are presented in **Figures 4A–C**. The fold change is in parenthesis. Taken together, the mean with range fold change of the inflammatory markers was 3.6 (2.5-4.4). The classical proinflammatory cytokines TNF, IL-1β, IL-6, and IL-8 are presented in **Figures 5A–D**. The fold change is in parenthesis. Except for IL-8, the mean and range fold change of these cytokines taken together was 4.3 (1.8-7.5). The regulatory cytokines and interferons, IL-10, IL-12p40, IFNα, and IFNγ are presented in **Figures 6A–D**. The fold change is in parenthesis. Taken together, the mean with range fold change was 3.7 (1.4-8.9).

### Biochemical and Organ Damage Markers

**Figure 7:**
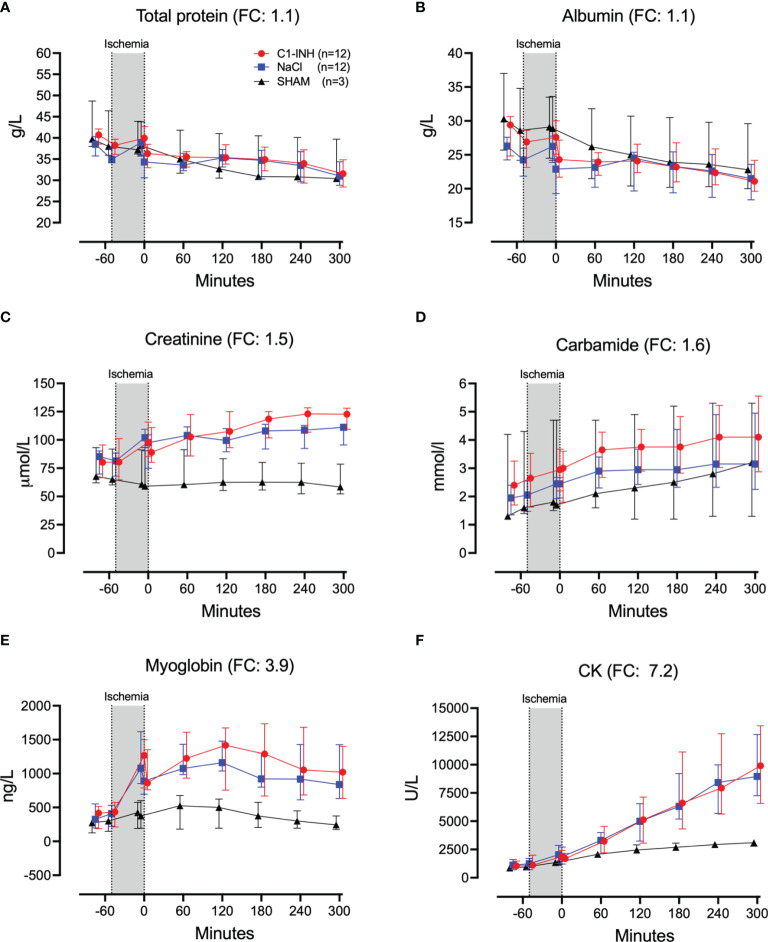
Biochemical and organ damage markers I. **(A)** Total protein, **(B)** albumin, **(C)** creatinine, **(D)** carbamide, **(E)** myoglobin, and **(F)** creatine kinase (CK) were measured in serum of 24 piglet undergoing clamping of the thoracic aorta at the Th8 level. Upfront 12 piglets received human saline intravenously and 12 received C1-INH (250 IU/kg), respectively. Three sham animals received thoracic opening without clamping. Reperfusion started at 0 minutes and lasted 5 hours. Longitudinal time data are expressed as median and interquartile range. “Ischemia” indicates the 45 minutes period of clamping. NaCl and C1-INH-groups were combined when there were no differences in the fold change (FC) calculation. FC is the change from before cross-clamp onset to maximum change.

**Figure 8:**
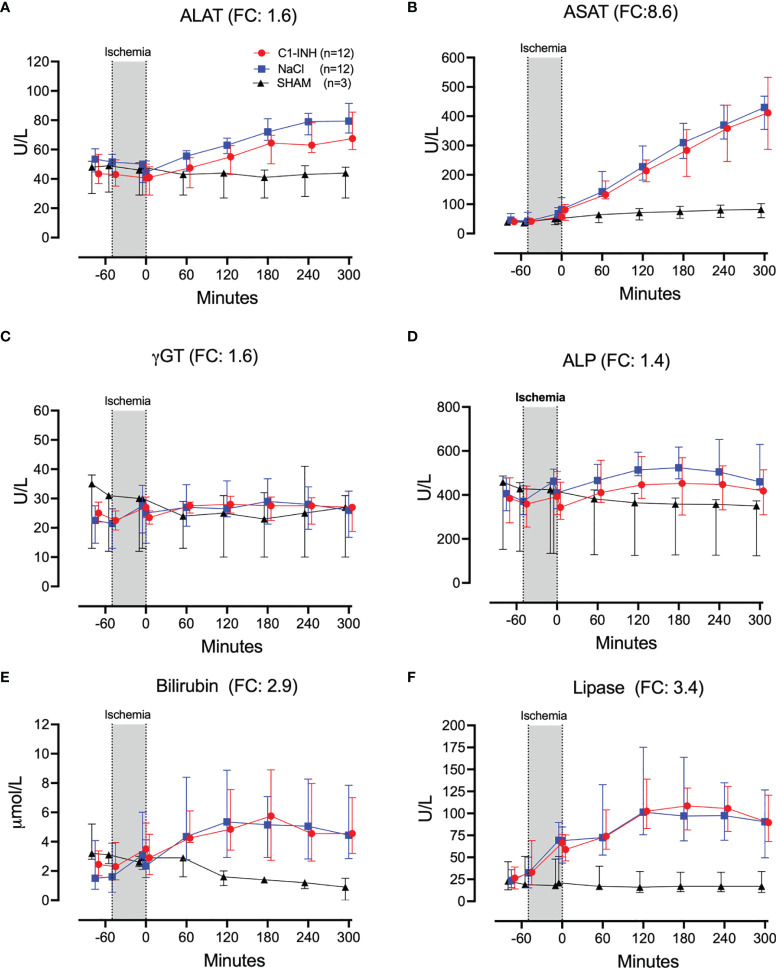
Biochemical and organ damage markers II. **(A)** Alanine aminotransaminase (ALAT), **(B)** aspartate aminotransferase (ASAT), **(C)** gamma-glutamyl transferase (γGT), **(D)** alkaline phosphatase (ALP), **(E)** bilirubin, and **(F)** lipase were measured in serum of 24 piglets undergoing clamping of the thoracic aorta at the Th8 level. Upfront 12 piglets received human saline intravenously and 12 received C1-INH (250 IU/kg), respectively. Three sham animals received thoracic opening without clamping. Reperfusion started at 0 minutes and lasted 5 hours. Longitudinal time data are expressed as median and interquartile range. “Ischemia” indicates the 45 minutes period of clamping. NaCl and C1-INH-groups were combined when there were no differences in the fold change (FC) calculation. FC is the change from before cross-clamp onset to maximum change.

Plasma proteins, kidney, and muscle markers were investigated. The absolute values and time course of total protein, albumin, creatinine, carbamide, myoglobulin, and CK are presented in **Figures 7A-F**. The fold change is in parenthesis. Taken together, the mean with range fold change was 2.5 (1.1-7.2). The following general and specific organ damage markers were measured: ALAT, ASAT, γGT, ALP, bilirubin, and lipase. The absolute values and time course are presented in **Figures 8A–F**. The fold change is in parenthesis. Taken together, the mean with range fold change was 3.2 (1.4-8.6).

### Cytokines in Tissue Analysis

**Figure 9:**
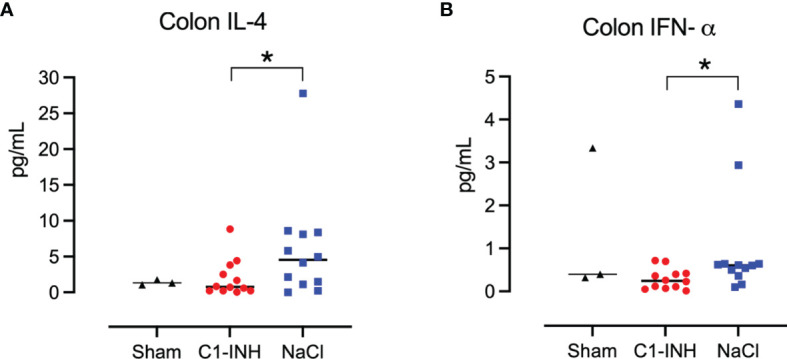
Cytokines in tissue. Post mortem biopsies from seven organs were homogenised and analysed for cytokines by multiplex immunoassays. C1-INH-treatment had no effect on the tissue cytokines except for two cytokines in the colon tissue: **(A)** interleukin (IL)-4 and **(B)** interferon (IFN)-α. Asterisks mean p=0.022 and p=0.016, respectively.

Eight organs were investigated for cytokines in postmortem biopsies. In seven organs there was no difference between the treated and untreated groups. In the colon, IL-4 and IFN-α showed a significant reduction in the C1-INH-treated group compared to the non-treated group (p=0.022 and 0.016, respectively) (**Figures 9A, B**).

## Discussion

The developed novel porcine model of ischemia-reperfusion injury after thoracic aorta cross-clamping produced robust results and proved to be a forceful and reliable model for the assessment of large scale IRI. The animals receiving thoracic aorta cross-clamping showed signs of severe large-scale ischemia with substantial lactic acidosis and, typical for porcine models, pulmonary hypertension. Keeping animals stable was challenging due to the severity of ischemia, but it was manageable. Despite severe hemodynamic changes, the animals in general responded in a uniform way. The effects of the IRI on blood and tissue markers also showed uniformity, supporting the value of the model and its potential for future testing of drugs aimed to restrict systemic IRI.

We did not see any significant effects of treatment with C1-INH on the damage from IRI in our model. Other animal studies have previously shown effect of C1-INH on IRI (19–22). These studies may not be fully comparable as few of them are in pigs, and most importantly do not involve a larger part of the body in a near systemic way. Our model is global and one of the most forceful models described, where all sub-diaphragmatic organs are virtually devoid of blood supply for 45 minutes. Therefore, the IRI in our model could be so severe that some inhibitors, including C1-INH, are inefficient.

Furthermore, blood in intercostal arteries and their branches could bypass the aortic clamping in some species. Also the aortic trifurcation in pigs could result in variable aortic retrograde flow compared to humans (23).Thus, interspecific differences could explain why human C1-INH did not improve outcome in our study. Finally, minor differences in the glycosylation of the C1-INH-protein could be of importance. An IRI study in mice found plasma-derived C1-INH, as we have used in the present study, inferior to recombinant human C1-INH (24). On the other hand, half-life of a recombinant human C1-Inhibitor produced in rabbits with a distinctive glycosylation (Ruconest, Pharming) had a shorter half-life in pigs than humans (14). However, the half-life of plasma-derived C1-inhibitor as we used in our study, administered both i.v. and subcutaneously to pigs had half-lives of more than 24 hours (25). Therefore C1-inhibitor levels are expected to remain high during our study period.

We have experienced in several *in vitro* studies that pharmacological inhibition of classical complement activation using C1-INH require extensively high doses, up to 20 times the physiological concentration (13). To the extent that the classical complement pathway plays an important role in IRI, this is in line with Ziccardi who 40 years ago found C1-INH to poorly inhibit when immunoglobulins were involved (26). Therefore, the dose of C1-INH per kg could have been insufficient to inhibit complement activation in severe IRI. Although some studies show effect of a lower (27) and some with higher doses up to 500 U/kg C1-INH (28), the reperfused organs were smaller, varying from skin flaps to kidneys and part of the heart. This makes a comparison of the studies difficult. In some models, researchers infuse C1-INH intra-arterially, directly into the targeted organs, increasing the dose pr kg tissue considerably. But even then, C1-INH can be inefficient. In a porcine infarction model Schreiber et al. found no effect of intracoronary infusion of 500 units C1-INH (29). However, the model by Schreiber et al. differed importantly from earlier models in that C1-INH was given after ischemia and during reperfusion. In our study C1-INH was infused prior to ischemia. Taken together, lack of effect in our global and forceful model due to too low C1-INH concentration cannot be totally excluded.

Supraphysiological doses of C1-INH have previously been reported to induce a procoagulant state which could have masked a positive effect by C1-INH (30). However, an *in vitro* study by our group found the opposite showing a dose-dependent anticoagulant effect of C1-INH in human whole blood (31). Our *in vivo* data from the present study also suggests a tendency towards an anticoagulatory effect, showing a non-significant tendency towards a reduction in TAT by C1-INH treatment. This could be explained by inactivation of FXII resulting in inhibition of the contact system by C1-INH, which could play a more significant role in IRI in this model. Cacci et al. suggested that thrombin inhibition by C1-INH might play a more important role in cases of severe inflammation as other major anticoagulatory systems are reduced in this state (32). Taken together, procoagulant effects of the C1-INH-infusion in the present study is an unlikely explanation for the observed lack of effect.

Publication biases could lead to overestimates of beneficial effects of C1-INH in IRI and underestimate negative results. According to Fanelli the proportion of positive results in the scientific literature in general increased to 85.9% in 2007 with a yearly increase of 6%, constant across most of the disciplines and countries (33). Therefore, our finding of limited effect of C1-INH in global ischemia is in our opinion an important contribution to the current literature. C1-INH is often placed on the list of complement inhibitors. Our study supports a general notion that C1-INH is not an efficient therapeutic, except for the reconstitution of patients with C1-INH deficiency. In our opinion, C1-INH should not be placed on the list of specific inhibitors for complement inhibition in the future, as we also have reviewed previously (34).

The present study raises the question which therapeutic targets could potentially reduce IRI.*in vivo* According to the danger model introduced by Matzinger, an inflammatory response involving innate immunity and complement can be expected in IRI (35). Therefore we anticipate a possible beneficial effect of combined inhibition of CD14 and complement C5 in IRI. In pediatric kidney transplant recipients, C5 inhibition with eculizumab prior to graft reperfusion was shown to improve early graft function, graft morphology and early graft survival indicating a reduction in IRI (36). There are also several other therapies available including highly specific complement inhibitors, as well as drugs targeting key upstream innate immune recognition molecules (34).

Our aim to establish a new porcine model for thoracic aortic cross-clamping using light weight piglets to allow for a minimal use of costly experimental therapeutics, while preserving translational value of the model is also a limitation of the study. The model is focused on the acute response and do not cover late complications.

## Conclusion

This study describes a novel, forceful model of porcine IRI that did not respond significantly to adequate C1-INH treatment but should allow for future testing of other drugs that could attenuate global IRI.

## Data Availability Statement

The original contributions presented in the study are included in the article/supplementary files. Further inquiries can be directed to the corresponding author.

## Ethics Statement

The animal study was reviewed and approved by Norwegian National Animal Research Authority (FOTS-ID-8197).

## Author Contributions

EN, YM, and TM designed the study. O-LB, JG, AD, HF, JL, KP, LR, RS and HJ planned and/or performed the experiments and analyses. EN and TM drafted the paper. EN and TM edited the draft. All authors critically revised the paper and approved the final draft.

## Conflict of Interest

The authors declare that the research was conducted in the absence of any commercial or financial relationships that could be construed as a potential conflict of interest.

## Publisher’s Note

All claims expressed in this article are solely those of the authors and do not necessarily represent those of their affiliated organizations, or those of the publisher, the editors and the reviewers. Any product that may be evaluated in this article, or claim that may be made by its manufacturer, is not guaranteed or endorsed by the publisher.
